# Maternal exposure to an environmentally relevant dose of triclocarban results in perinatal exposure and potential alterations in offspring development in the mouse model

**DOI:** 10.1371/journal.pone.0181996

**Published:** 2017-08-09

**Authors:** Heather A. Enright, Miranda J. S. Falso, Michael A. Malfatti, Victoria Lao, Edward A. Kuhn, Nicholas Hum, Yilan Shi, Ana Paula Sales, Kurt W. Haack, Kristen S. Kulp, Bruce A. Buchholz, Gabriela G. Loots, Graham Bench, Kenneth W. Turteltaub

**Affiliations:** 1 Biosciences and Biotechnology Division, Lawrence Livermore National Laboratory, Livermore, CA, United States of America; 2 Center for Accelerator Mass Spectrometry, Lawrence Livermore National Laboratory, Livermore, CA, United States of America; 3 Data Analytics and Decision Sciences, Lawrence Livermore National Laboratory, Livermore, CA, United States of America; University of Louisville School of Medicine, UNITED STATES

## Abstract

Triclocarban (TCC) is among the top 10 most commonly detected wastewater contaminants in both concentration and frequency. Its presence in water, as well as its propensity to bioaccumulate, has raised numerous questions about potential endocrine and developmental effects. Here, we investigated whether exposure to an environmentally relevant concentration of TCC could result in transfer from mother to offspring in CD-1 mice during gestation and lactation using accelerator mass spectrometry (AMS). ^14^C-TCC (100 nM) was administered to dams through drinking water up to gestation day 18, or from birth to post-natal day 10. AMS was used to quantify ^14^C-concentrations in offspring and dams after exposure. We demonstrated that TCC does effectively transfer from mother to offspring, both trans-placentally and via lactation. TCC-related compounds were detected in the tissues of offspring with significantly higher concentrations in the brain, heart and fat. In addition to transfer from mother to offspring, exposed offspring were heavier in weight than unexposed controls demonstrating an 11% and 8.5% increase in body weight for females and males, respectively. Quantitative real-time polymerase chain reaction (qPCR) was used to examine changes in gene expression in liver and adipose tissue in exposed offspring. qPCR suggested alterations in genes involved in lipid metabolism in exposed female offspring, which was consistent with the observed increased fat pad weights and hepatic triglycerides. This study represents the first report to quantify the transfer of an environmentally relevant concentration of TCC from mother to offspring in the mouse model and evaluate bio-distribution after exposure using AMS. Our findings suggest that early-life exposure to TCC may interfere with lipid metabolism and could have implications for human health.

## Introduction

Triclocarban (TCC) is an antimicrobial found in many personal care products including deodorants and antibacterial soaps [1], and is a common contaminant in wastewater [2]. TCC has been detected in U.S. water at a mean concentration of 670 picomolar (pM, 213 ng/L) with maximum concentrations in the nanomolar range (6750 ng/L) [2]. With the widespread occurrence of TCC in the environment, bioaccumulation has been observed in organisms including earthworms, algae, European starling eggs, plants and snails [3, 4] and reproductive effects have been noted as a result of low level exposure [5, 6].

At higher concentrations, deleterious reproductive effects have been observed in rodents; evidence of endocrine disruption and lower testes/body weight ratios have been shown in rats (~170mg/kg/d) [7] and reduced offspring survival has been noted for rats when exposed to TCC in their chow (0.2–0.5% w/w) during lactation [8]. Interaction with endogenous hormones to potentially affect development of sex organs and interfere with reproduction has also been reported [9–12]. In cells, exposure to TCC at micromolar concentrations has been shown to enhance estradiol or testosterone dependent activation of estrogen and androgen responsive genes [9]. This amplification effect was also demonstrated in castrated rats where administration of TCC and testosterone increased all male sex accessory organs [10]. More recently, at environmentally relevant concentrations (nM), TCC has been shown to exert estrogenic activity in kidney cells [11]. Therefore, due to potential risk for human exposure, TCC has raised concerns regarding its effects on human health.

Exposure to endocrine disrupting compounds (EDC), such as TCC during development may pose a serious health risk to the developing embryo and fetus, as they are more sensitive to perturbations in hormone levels which may result in changes that are often irreversible [13]. Additionally, protective mechanisms that are present in adults such as the blood-brain barrier, liver metabolism, detoxifying enzymes, DNA repair mechanisms and a competent immune system are not fully functional during development. These deficits along with an increased metabolic rate may make the developing organism more susceptible to damage induced by exposure to EDCs [14]. In humans, TCC has been detected (μg/L) in both urine (86.7%) and cord blood (22.9%) of mother/child pairs from Brooklyn, New York [15]. The potential risk of TCC exposure during development and the contribution of exposure to diseases developed later in life have not yet been thoroughly investigated, especially at concentrations that humans commonly encounter in their environment. To date, there are no data on the effects of TCC exposure at environmentally relevant concentrations, its ability to exchange between mother and offspring during development, and consequently the potential risk posed by this exposure during development to human health.

The aim of this study was to determine whether an environmentally relevant concentration of TCC transferred from mother to offspring through consumption of contaminated drinking water. We investigated whether TCC (100 nM) crossed the placental barrier, and given the fat solubility of TCC, investigated whether TCC could transfer from mother to offspring during neonatal exposure through breast milk. Accelerator mass spectrometry (AMS), an ultrasensitive measurement technique that measures rare, long-lived isotopes [16] at attomolar (amol) concentrations was used in this study to quantify ^14^C-TCC transfer to offspring after exposure through gestation day 18 (GD18) and after lactational exposure through postnatal day 10 (PND10). The sensitivity of AMS enables the long-term study of environmentally relevant concentrations of potentially toxic compounds. In a long-term study, TCC exposed neonatal offspring were carried out to six weeks to investigate whether neonatal exposure had an effect on offspring through maturity.

## Materials and methods

### Chemicals

^14^C-Triclocarban (TCC) was obtained from Moravek Biochemicals (Brea, CA) with a specific activity of 1.11x10^12^ Bq/mol (30 mCi/mmol) and a radiochemical purity of 98.8%. TCC was dissolved in dimethyl sulfoxide (DMSO) prior to dilution into drinking water; the percent of DMSO in drinking water was less than 0.001%. Water aliquots for dose determination were added to Universol cocktail (VWR, International, Radnor, PA) before scintillation counting.

### Bottle preparation and stability

^14^C-TCC demonstrated high adsorption in traditional animal water bottles synthesized of rubber and plastic materials; 40–70% of the initial dose was lost within hours. Therefore, custom-made stainless steel water bottles were used for all water dosing studies. Stability of 100nM ^14^C-TCC in water was tested (n = 2) for these bottles over two weeks with liquid scintillation counting (LSC) (Perkin Elmer). An aliquot from each bottle was counted at the initial time point (1 hour) and each subsequent time point; data is expressed as percent of activity lost (S1 Fig). Based on the average loss observed (~30%), 130 nM ^14^C-TCC water bottles were prepared for all *in vivo* exposures (1.04 nCi/mL). Bottles were stabilized for two days before dosing; once administered to animals, bottles were changed every 4–5 days. For each dosing bottle, an aliquot was taken before and after administration and counted with LSC. While in use, bottles were weighed to estimate water consumption by each dam. Both consumption amount and ^14^C-TCC activity measured by scintillation counting were used to estimate ingested dose (ID).

### Animals

All animal experiments were conducted following the guidelines and regulations set by Lawrence Livermore National Laboratory, including IACUC approval. CD-1 female (8–10 weeks) and male mice (10–12 weeks) were used for all studies (Harlan, Livermore, CA). Mice were housed individually in polystyrene cages containing hardwood bedding and kept on a 12 h light/dark cycle in a ventilated room maintained at 24° C. Food and water were provided *ad libitum*.

### ^14^C-TCC dosing and sample collection

**Fig 1:**
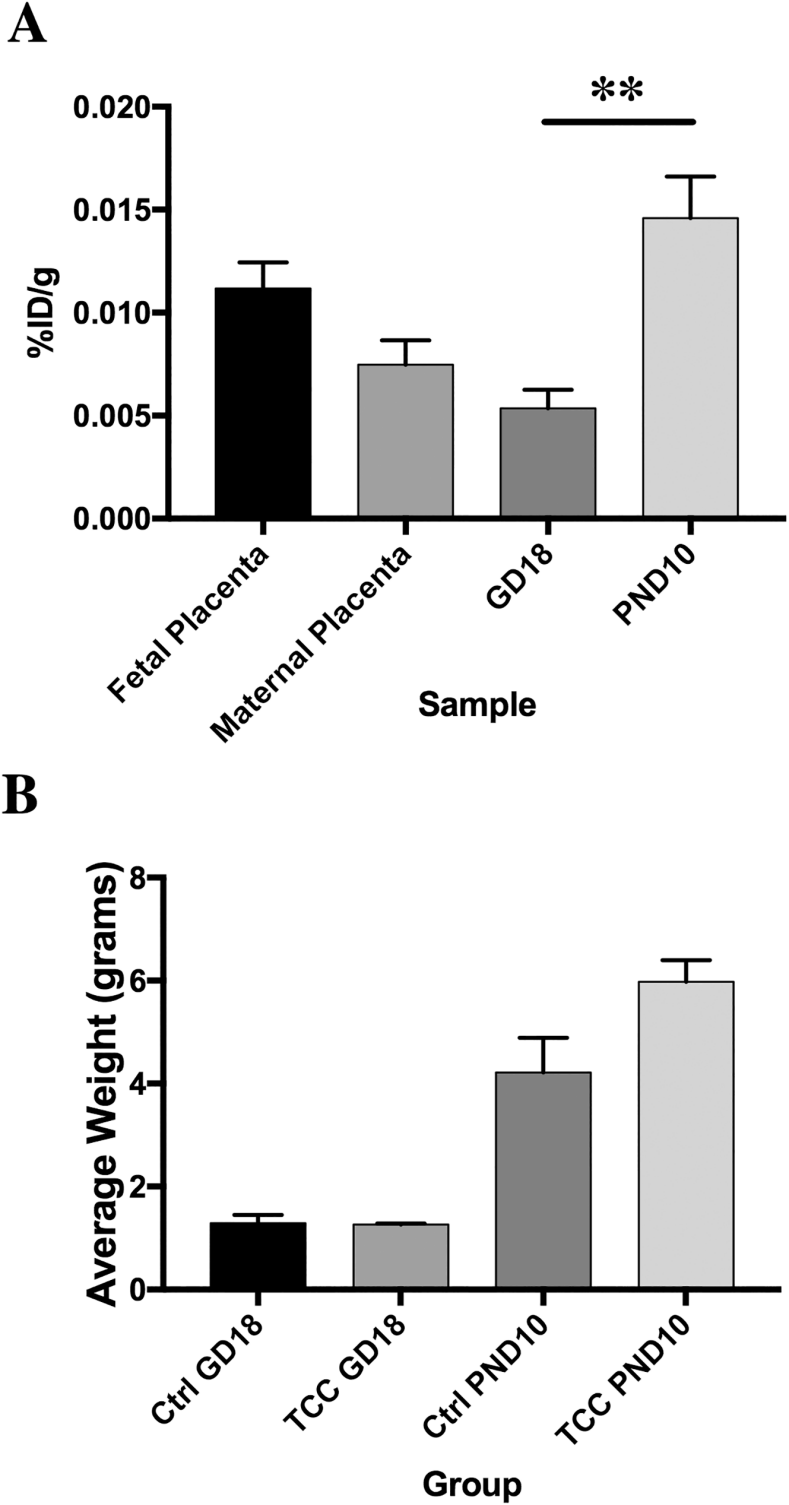
Effects of exposure to TCC during gestation and lactation. (A) TCC levels in fetal and maternal placental tissues and fetus at gestation day 18 (GD18) (n = 6 litters), and offspring after lactation at postnatal day 10 (PD10) (n = 8 litters). Data is expressed as the average percent ingested dam dose/gram tissue (%ID/g) ± SEM with AMS measurements for homogenates of each individual litter. (B) Average offspring weight for control and TCC exposure groups GD18 (Ctrl, n = 6 litters, TCC, n = 6 litters) and PND10 (Ctrl, n = 2 litters, TCC, n = 8 litters). Data is expressed as the mean ± SEM with the litter as the unit. **p<0.01 when comparing GD18 and PND10.

For TCC exposures, females were housed with males (4 females, 1 male per cage) and monitored daily for vaginal plugs (gestation day 0). For *in utero* exposure, dams were given ^14^C-TCC drinking water starting on the day of plug visualization through gestation day 18 (GD18). At GD18, dams were euthanized by CO_2_ asphyxiation. Fetuses were removed and separated from the placenta, rinsed three times in 1X PBS and stored at -80°C until analysis. Maternal and fetal placental tissues were isolated on ice, rinsed three times in 1X PBS and stored similarly to fetal tissue. The numbers of fetuses were 11.67 ± 1.99 for the control group (n = 6 litters) and 15.17 ± 1.11 (n = 6 litters) for the TCC group; no significance was determined for litter sizes between groups (p = 0.15). For the lactation exposure group, dams were given standard drinking water until litters were born (Day 19/20). On the day of litter delivery, ^14^C-TCC drinking water was given to dams for the first 10 days of lactation. On postnatal day 10 (PND10) offspring were euthanized, weighed and rinsed three times in 1X PBS before storing at -80°C until AMS analysis. The numbers of offspring were 21.50 ± 2.5 for the control group (n = 2 litters) and 12.88 ± 1.20 for the TCC group (n = 8 litters). For each exposure group, control dams with standard drinking water were run in parallel for comparison. Due to significant differences in litter sizes for the lactation exposure group (p = 0.01), litter was used as the unit for Fig 1 (GD18, PND10 groups); mean body weights were calculated for each litter.

For the long-term lactation study, dams were dosed with ^14^C-TCC drinking water similar to the lactation exposure described above, starting on the day of litter delivery to PND10. On PND10, dams were administered standard drinking water through weaning. An untreated control group was run in parallel for comparison. The numbers of offspring were 11.60 ± 0.4 (n = 5 litters) for the control group and 11.83 ± 0.75 (n = 6 litters) for the TCC treated group. Offspring were weighed every five days starting at postnatal day 1. At PND21, offspring were separated into males and females and sex specific body weights were measured through PND56. A subset of offspring (n = 20 for TCC, n = 20 control) was sacrificed at PND42 for bio-distribution analysis using AMS and gene expression; these offspring were randomly chosen from all litters in both groups. At PND56, the remaining offspring were euthanized after weighing. Tissues (liver, spleen, kidney, lungs, heart, brain, inguinal fat, gonadal/ovarian fat, retroperitoneal fat, uterine fat, uterus, muscle, gastrointestinal tract, colon, adrenals, thymus, ovaries and testes) were collected from all offspring and stored at -80° C until analysis. Dams were euthanized on the day of offspring weaning (PND21). Tissues (as above) were collected and stored at -80° C until AMS analysis. Data were analyzed with the unit as the individual offspring for this study; litter sizes between groups were not significant (p = 0.80). Peirce’s criterion was used to eliminate outliers [17].

### AMS analysis

All tissues for AMS analysis were homogenized prior to analysis using a previously established method [18]. Samples were incubated in 1–2 ml of collagenase buffer overnight at 37°C with gentle agitation; after digestion, samples were vortexed to break up the tissue in solution. Plasma samples were analyzed neat, no digestion was necessary. A small aliquot of each sample (10–100 ±l, depending on tissue carbon content) was used for analysis. For litter analyses (Fig 1A) samples were pooled to analyze a representative sample for each litter. For tissue specific organ analyses, each tissue from each offspring and dam was measured. Prior to AMS analysis, samples were converted to graphite as previously described [19]. Each measured ^14^C/C ratio for tissues was converted to pmol of TCC/g of tissue or ml of plasma using the specific activity of TCC and the carbon content of the tissue (10–15% for tissue, 3.8% plasma) as described previously [20]. Peirce’s criterion was used to eliminate outliers [17] in this data set.

For high performance liquid chromatography analysis of TCC content in offspring, representative samples from three litters were used after exposure through PND10. Homogenized samples were extracted with 50/50 methanol/acetone added to samples (5:1) for 24 hours with agitation [4]. Samples were then centrifuged for 30 minutes at 800 x g. The resulting supernatant was dried under vacuum using a Savant Speedvac SPD2010 (Thermo Scientific) and resuspended in 0.5mL of nanopure water. Analytes were extracted using 60mg 0.33 ±m polymeric reversed phase cartridges (Strata-X, Phenomenex). Cartridges were preconditioned with 1 mL of methanol and 1 mL of nanopure water. Samples were then passed through and cartridges were washed with 1 mL of 5% methanol. Analytes were then eluted using 1 mL of acetonitrile. The resulting solutions were then dried under vacuum and reconstituted in 20 ±l of acetonitrile. UPLC analysis was performed using a Waters Acquity system (Waters, Milford, MA). Separation was performed on a BEH C18 column with dimensions of 2.1 X 50 mm and a particle size of 1.7 ±m. A binary gradient with a flow rate of 0.2 mL/min of water was used. Mobile phase A consisted of nanopure water and mobile phase B was 100% acetonitrile. The gradient was as follows: B = 2% for 11 min which increased to 100% by 12 minutes. The eluent was collected in one minute fractions and each fraction was analyzed for ^14^C content by AMS utilizing 1 ±L of tributyrin delivered in capillary tubes as the carbon carrier to bring the carbon content to 0.6 mg/sample (necessary for efficient conversion of samples to graphite for AMS measurement).

### Quantitative real-time PCR analysis

For gene expression analysis, representative offspring were randomly selected across five litters at PND42 for both male and female TCC treated and control litters from the long-term lactation exposure study as described above. Total RNA from liver and adipose tissues was extracted from PND42 offspring tissue homogenates in Qiazol lysis reagent (Qiagen) using RNeasy minikit according to the manufacturer's protocol. Reverse transcription was performed using SuperScript III Reverse Transcriptase (Life Technologies) according to the manufacturer's protocol. Quantitative real-time PCR analysis (qPCR) was then performed on the resulting cDNA with SYBR Select Master Mix (Life Technologies) on an Applied Biosystems *7900HT* Fast Real-Time PCR System (Applied Biosystems) with primers listed in Table 1. Quadruplicate biological samples were analyzed per condition and each sample was analyzed from triplicate reactions. All qPCR data was analyzed using the comparative CT method (ΔΔCT) for relative quantification and β-Actin was used as the reference gene.

**Table 1:** Primers used for qPCR analysis.

Gene	Gene Abr	Forward Primer	Reverse Primer
PPARalpha	Ppara	TACTGCCGTTTTCACAAGTGC	AGGTCGTGTTCACAGGTAAGA
CPT1A	CPT1A	AGATCAATCGGACCCTAGACAC	CAGCGAGTAGCGCATAGTCA
CPT2	CPT2	CAGCACAGCATCGTACCCA	TCCCAATGCCGTTCTCAAAAT
TNFalpha	Tnf	CTGAACTTCGGGGTGATCGG	GGCTTGTCACTCGAATTTTGAGA
leptin	Lep	GAGACCCCTGTGTCGGTTC	ATACCGACTGCGTGTGTGAAATG
adiponectin	Adipoq	TGTTCCTCTTAATCCTGCCCA	CCAACCTGCACAAGTTCCCTT
PPARgamma	Pparg	CTCCAAGAATACCAAAGTGCGA	GCCTGATGCTTTATCCCCACAGAC
SREBP-1c	Srebf1	CAAGGCCATCGACTACATCCG	CACCACTTCGGGTTTCATGC
Actin	ActB	ACCTCTATGCCAACACAGTGC	CTGGAAGGTGGACAGTGAGG

### Hepatic triglyceride analysis

Liver tissue from representative offspring at PND56 was randomly selected across 4–5 litters for both male and female TCC treated and control litters. Liver triglycerides were measured in duplicate with a commercially available colorimetric kit (Cayman Chemical, Ann Arbor, MI). Liver tissue was prepared for analysis by mincing and mechanically homogenizing tissue (200mg) in the standard diluent buffer containing protease inhibitors (Sigma Aldrich). Homogenates were centrifuged at 10,000 x g for 10 minutes at 4°C. The resulting supernatant was stored at -80°C until analysis. Samples were diluted 1:5 with diluent buffer; each sample (10μl) and standard was run in duplicate. Absorbance was read at 540nm using a Spectramax Plus 384 microplate reader (Molecular Devices).

### Statistical analysis

Data is expressed as the average value ± standard error of the mean (s.e.m.). Peirce’s criterion was used to eliminate outliers [17] and is noted where used. Statistical analysis was performed using a student’s t-test (two-tailed) unless otherwise indicated; litter was used as the fundamental unit of comparison when appropriate and is noted in the experimental methods. The effect of TCC on offspring weight gain over time was analyzed via a hierarchical mixed effect statistical model (Laird and Ware 1982). Additional details for this statistical analysis is in S1 Appendix. A *p-*value of <0.05 was considered significant for all statistical tests.

## Results

We first investigated whether an environmentally relevant concentration of TCC could transfer from mother to fetus when the dams were exposed through drinking water up to gestation day 18 (GD18). Subsequently transfer of TCC to offspring through lactation was assessed when dams were exposed through drinking water from offspring birth to postnatal day 10 (PND10). ^14^C-TCC concentrations in litter homogenates were quantified using AMS. TCC was detected at low concentrations in offspring for both *in utero* and lactation exposure groups (Fig 1A). At GD18, 0.005% ± 0.001 of the ingested dose per gram (%ID/g) was detected in the fetus; higher concentrations were present in fetal (0.011%ID/g ± 0.001) and maternal placenta tissue (0.007%ID/g ± 0.001) confirming translocation across the placental barrier. A 3-fold higher concentration of TCC was detected in offspring homogenates at PND10 after exposure solely through lactation (0.015% ID/g ± 0.002, p = 0.003) compared to the *in utero* exposure demonstrating that TCC transfers readily through breast milk. We also observed a ~33% increase in body weight for the PND10 lactation exposure group relative to controls (Fig 1B), although this increase was not statistically significant. At PND10, TCC exposed offspring weighed 5.98 g ± 0.42 (n = 8 litters, p = 0.09) and their control counterparts weighed 4.22 g ± 0.67 (n = 2 litters). No significance differences (p = 0.89) in fetus weight were observed for the GD18 *in utero* TCC exposed group (1.26 g ± 0.02, n = 6 litters) when compared to controls (1.29 g ± 0.17, n = 6 litters) (Fig 1B).

**Fig 2:**
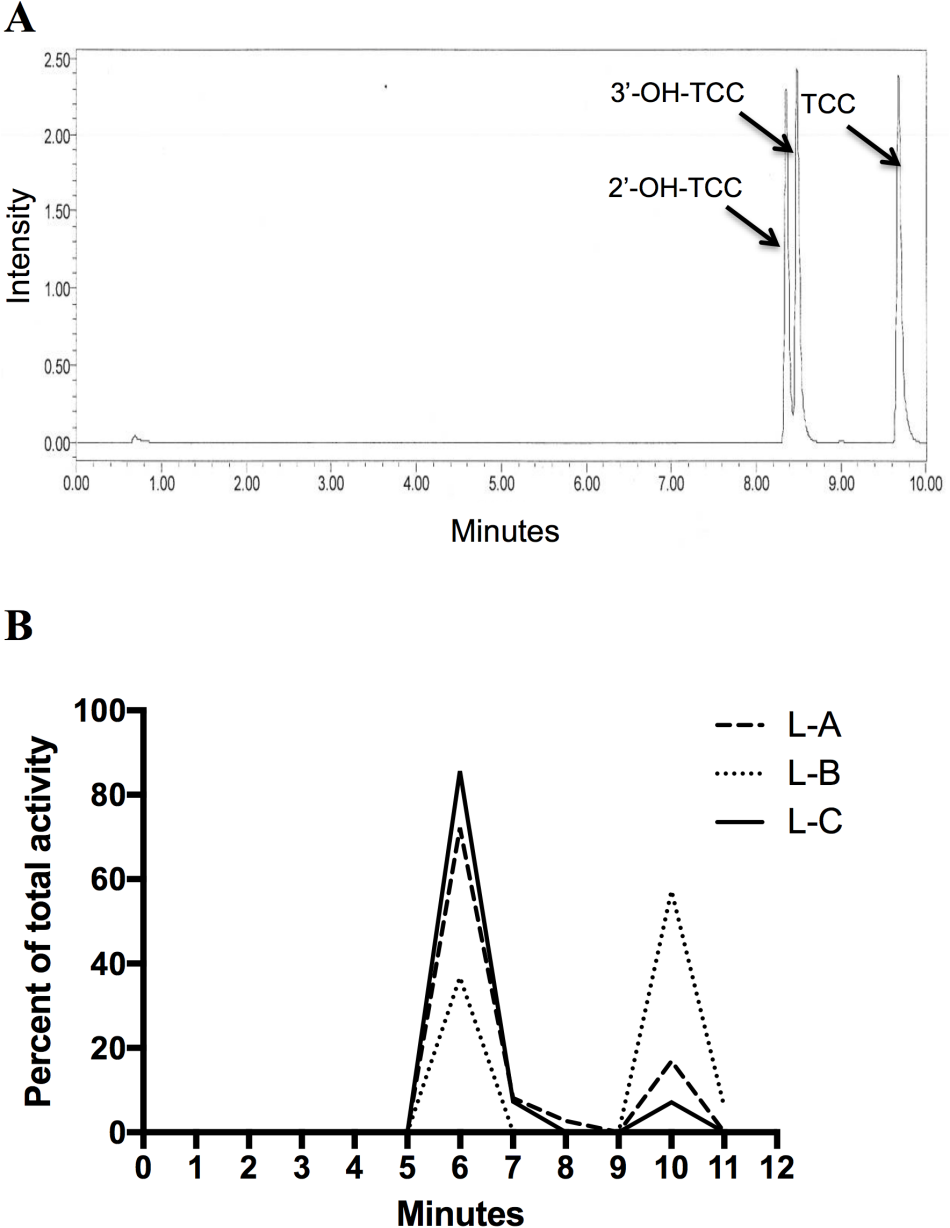
Ultra-performance liquid chromatography analysis (UPLC) of PND10 offspring. (A) UPLC Chromatogram of an authentic TCC standard and hydroxylated-TCC metabolite standards. TCC retention times: TCC = 10 min., 2’-OH-TCC = 8.4 min, 3’-OH-TCC = 8.6 min. (B) UPLC radio-chromatogram of extracted ^14^C-TCC from lactation exposed PND10 offspring homogenate. Data represents 3 individual samples run separately.

To identify whether the signal measured by AMS was from parent ^14^C-labeled TCC, we analyzed representative offspring homogenates from our PND10 lactation group using ultra-performance liquid chromatography (UPLC). A chromatogram showing the retention times for a TCC standard and the major hydroxylated metabolites, 2’-OH-TCC and 3’-OH-TCC, is shown in Fig 2A. The radio-chromatogram for extracted analytes from offspring homogenates is shown in Fig 2B. From three representative litter homogenates, the peak eluting at 10 min accounted for 29.07% ± 17.29% of the total recovered ^14^C activity and had the same retention time as the TCC authentic standard. The peak at a retention time of 6 min accounted for 70.93% ± 17.29% of the ^14^C activity but did not correspond to TCC or the 2 major hydroxylated metabolites 2’-OH-TCC or 3’-OH-TCC. This peak is presumably a glucuronide conjugate of OH-TCC since it has been shown that 2’-OH-TCC and 3’OH-TCC readily undergo uridine-5’-diphosphate-glucuronosyltransferase catalyzed glucuronidation to form an O-glucuronide-TCC conjugate for renal and biliary elimination [21].

**Fig 3:**
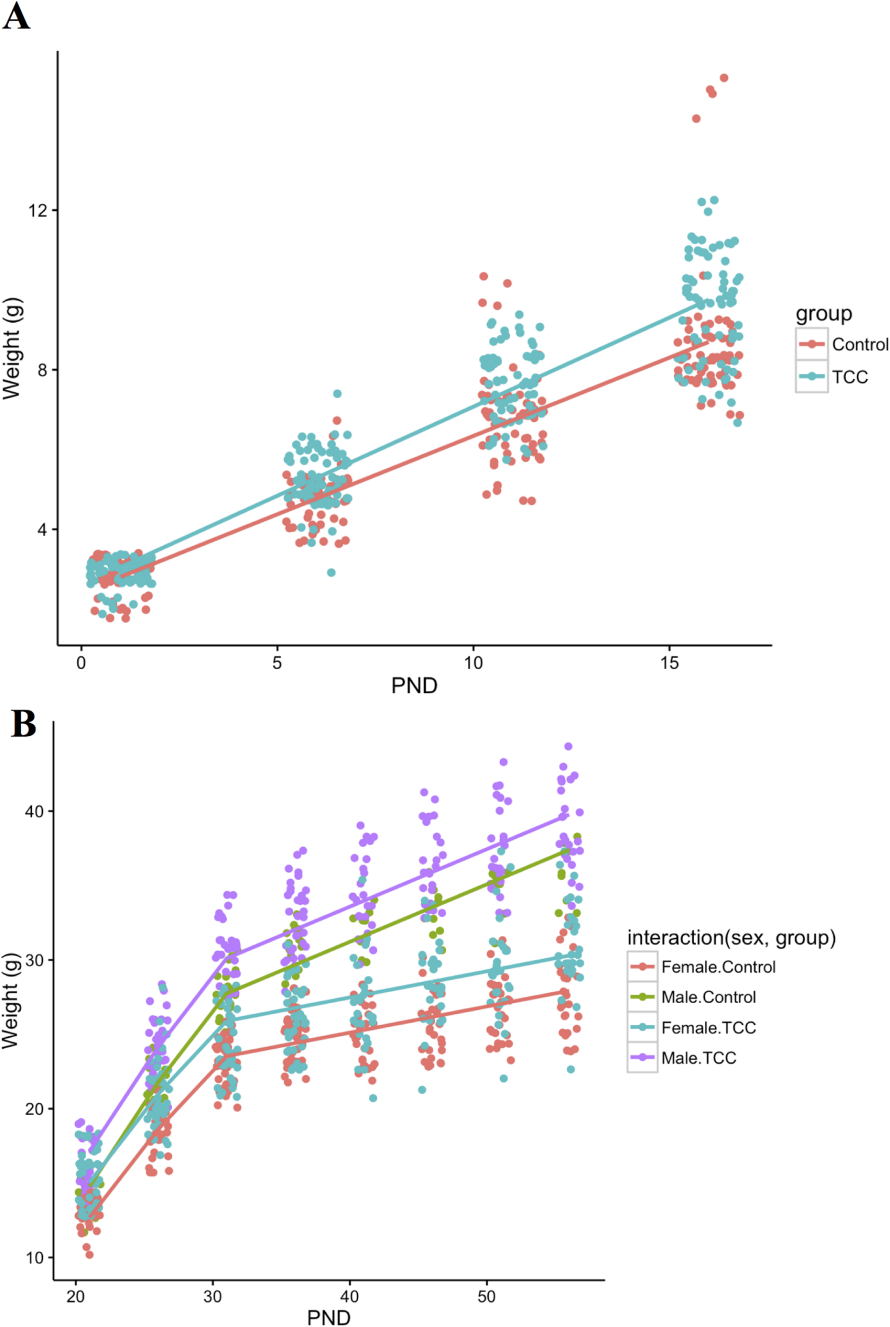
Body weights of offspring after exposure to TCC through lactation via ^14^C-TCC spiked dam drinking water (100 nM TCC). (A) Change in body weight of all offspring from birth through weaning (postnatal day 20). Red denotes control, and green TCC-exposed offspring. (B) Changes in body weight from PND20 through PND 56. Each color denotes a different combination of sex and experimental group. In both panels, points are the observed values and lines are unconditional (population-level) predictions for the corresponding categories. Control males, n = 18 offspring, TCC males, n = 34 offpsring, Control females, n = 40 offspring, TCC females, n = 37 offspring. The rate of weight gain from birth to weaning (PND21) was significantly higher for the TCC exposed group (p = 2.34E-5). Additionally, TCC exposed offspring from PND21-56 were significantly greater in weight compared to controls (p = 0.016).

Given the observed weight increase at PND10 for offspring exposed to TCC during lactation, we examined a second group of TCC-exposed offspring through eight weeks of age to determine whether elevated body weight persisted through maturity, and whether neonatal TCC exposure affected organ development. Offspring were exposed to TCC during lactation from birth to PND10. From birth through weaning (PND21), both baseline weight (p = 0.07) and the rate of weight gain (p = 2.34E-5) for the TCC exposed offspring were higher than that of the control group (Fig 3A). For PND21-56, TCC exposed offspring had a statistically significant increase in weight (p = 0.016) compared to the control group (Fig 3B).

**Fig 4:**
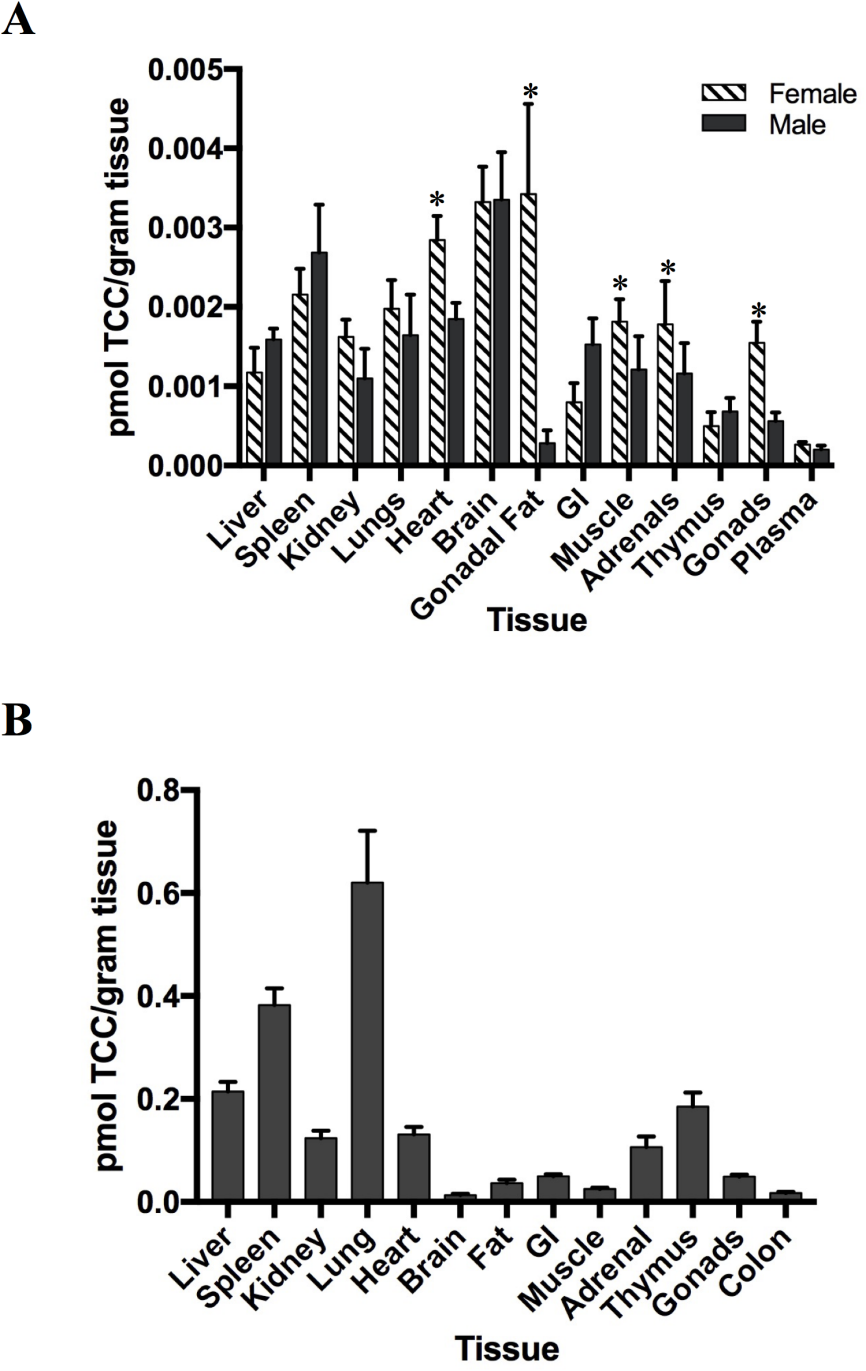
Tissue distribution of ^14^C-TCC in offspring and dams. Offspring bio-distribution (A) after lactational exposure, and bio-disribution in dams (B) after exposure via ^14^C-TCC spiked dam drinking water (100 nM). TCC concentrations were measured at PND42 in offspring and at PND21 (weaning) for dams. Data is expressed as pmol TCC per gram of tissue ± SEM. Offspring (n = 5); dams (n = 6). *p<0.05, when comparing females to males.

To determine TCC tissue distribution, harvested tissues were homogenized and ^14^C content was quantified in each tissue homogenate with AMS. ^14^C was found in all organs examined (Fig 4A) for both male and females at postnatal day 42 (PND42). Higher concentrations were detected in brain tissue compared to other tissues assayed with approximately 14% and 18% of total recovered ^14^C concentration found in female and male offspring brain tissues, respectively. Significantly higher ^14^C concentrations (*p*<0.05) were detected in female offspring’s gonadal fat, gonads, muscle and heart tissue compared to littermate males.

The bio-distribution of TCC in the dams exposed during lactation was also determined at offspring weaning (PND21). TCC and its metabolites were primarily distributed to major organs such as the lung, liver, spleen, heart and kidney; approximately 75% of total recovered ^14^C concentrations were localized in these organs (Fig 4B). In relation to these organs, ^14^C concentrations in the brain (<1%), muscle (~1%) and heart (~7%) of exposed mothers were much lower, unlike the higher percentages found in tissues of exposed offspring (14–18%, ~7%, and ~12% respectively).

**Fig 5:**
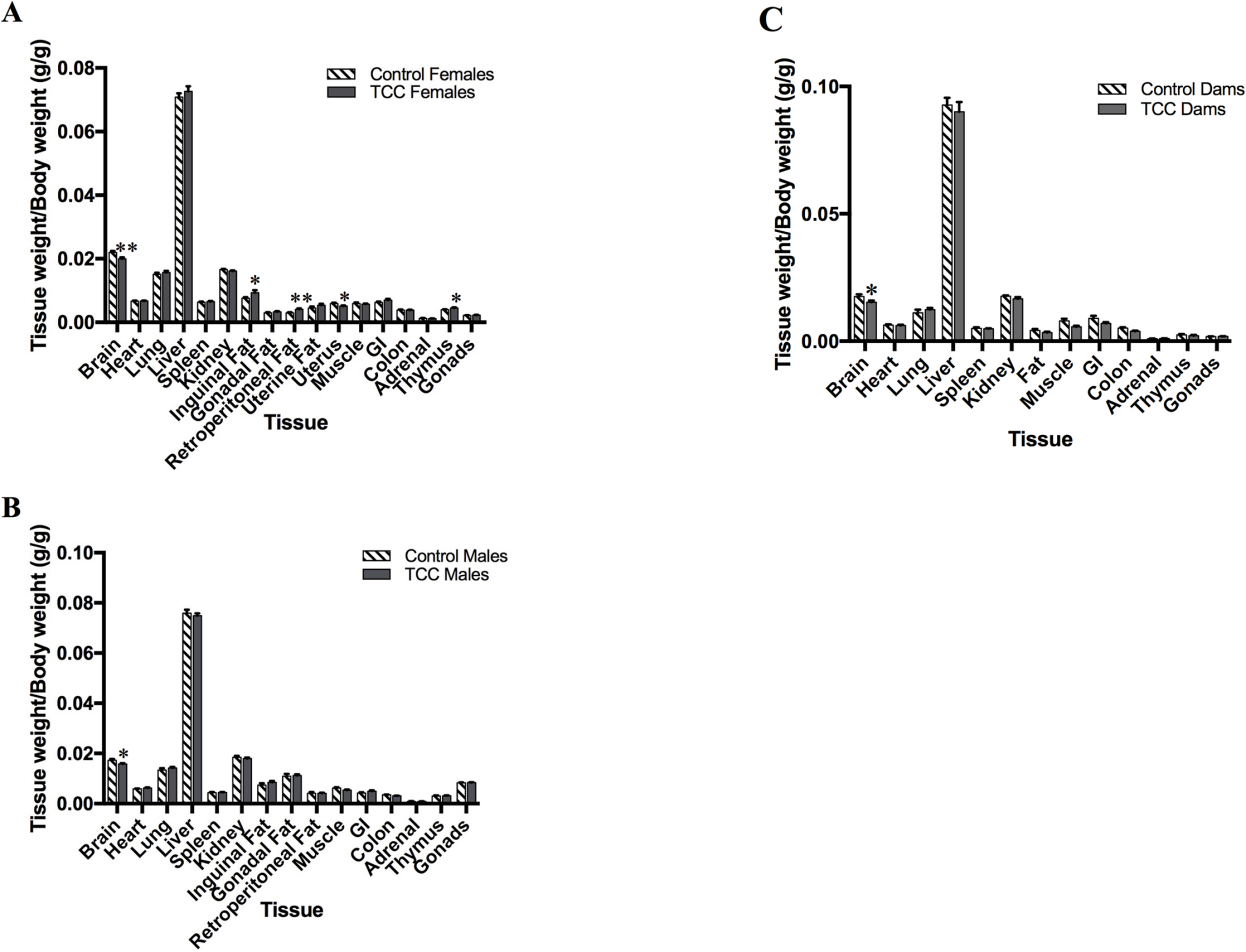
Tissue weight/body weight comparisons between control and TCC exposure groups. (A) Female offspring after lactational exposure at PND56 (Control n = 30; TCC females n = 27) (B) Male offspring after lactational exposure at PND56 (Control males, n = 9, TCC males, n = 24). (C) Dams after TCC exposure via ^14^C-TCC spiked drinking water (100 nM) (Ctrl, n = 6, TCC, n = 6). Data is expressed as the mean ± SEM *p<0.05, **p<0.01.

The brain, muscle, heart and fat are all organs involved in lipid metabolism and energy homeostasis [14, 22, 23]. Given the higher accumulation of ^14^C in these tissues and the overall greater body weights observed for TCC offspring, we investigated whether changes in specific organ masses also occurred in TCC treated offspring. Total organ weights normalized per unit body weight were analyzed for all groups of offspring at postnatal day 56 (PND56) (Fig 5A and 5B) and exposed dams (Fig 5C). Common for all TCC exposed groups was a reduction in brain size. A reduction of approximately 9% (p = 0.01 males, p = 0.005 females) of brain weight was noted in all offspring, while a ~13% reduction was found in exposed dams (p = 0.03). Uterine weight was reduced in TCC female offspring by ~14% (p = 0.02). Individual fat pads were collected to investigate whether TCC exposure had an effect on fat mass. Significance was found for the female group only with both inguinal and retroperitoneal fat having, on average, 22.97% (p = 0.0.043) and 39.66% (p = 0.001) greater mass respectively, compared to controls (Fig 5A). Additionally, a 13.56% increase in thymus weight was noted for female offspring (p = 0.02).

**Fig 6:**
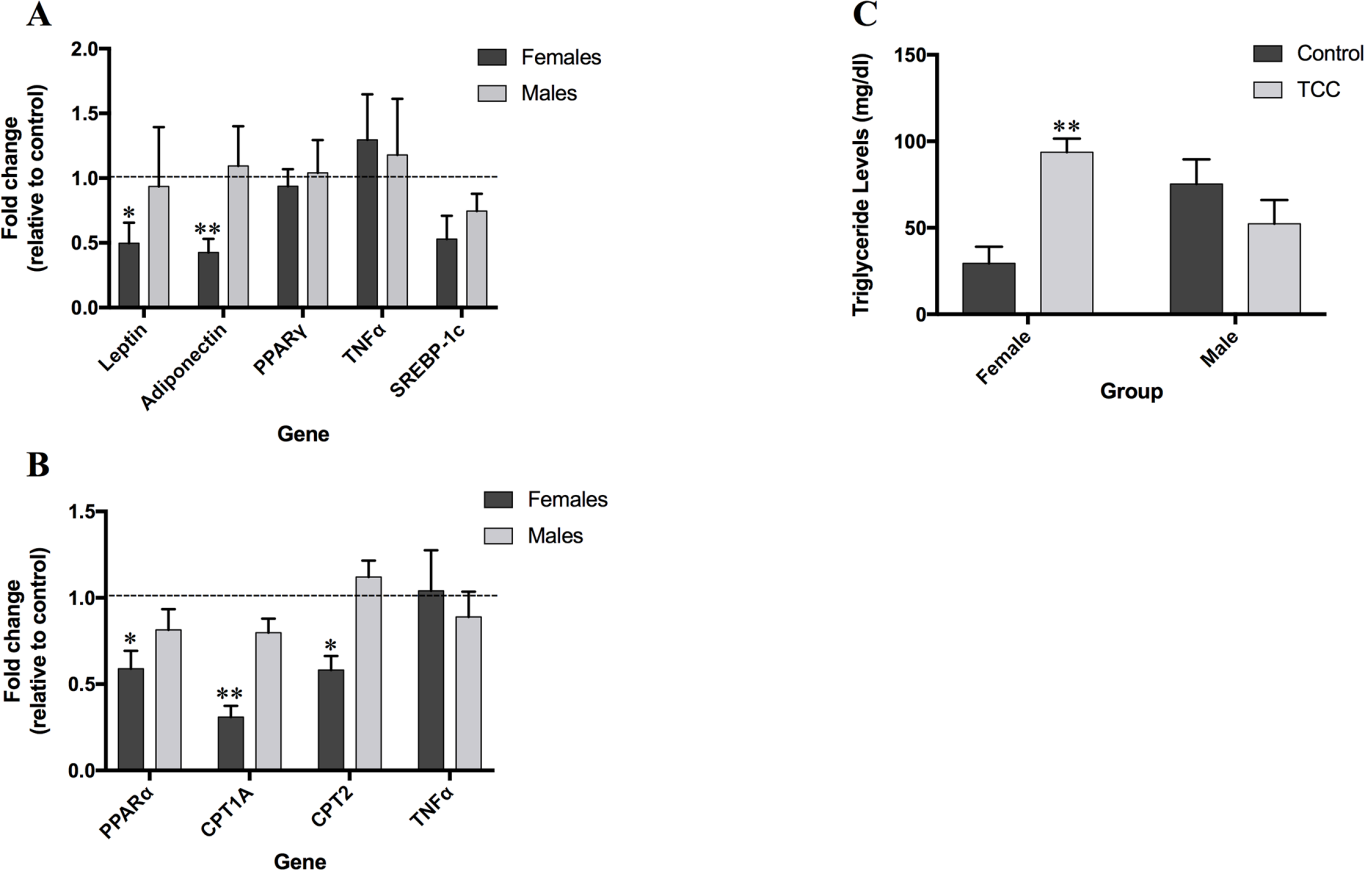
Gene expression changes in fat and liver tissue after 100nM TCC exposure. Expression changes relative to control in adipose tissue (A) and liver tissue (B) for male and female offspring at PND42 (n = 4). (C) Hepatic triglyceride concentrations for male and female offspring at PND56 (Ctrl female, n = 4, TCC female, n = 5, Ctrl male, n = 5, TCC male, n = 4). Data is expressed at the mean ± SEM. *p<0.05, **p<0.01, ^**#**^p<0.001 when comparing to controls.

Other endocrine disrupting compounds have been associated with increases in offspring weight and have been shown to disrupt lipid metabolism [14, 24, 25]. To determine whether TCC exposure and increased fat pad weights correlate with alterations in genes involved in lipid metabolism, we quantified the mRNA expression levels of genes involved in lipid metabolism in adipose and liver tissue using qPCR at PND42 (Fig 6). Significant down-regulation of genes involved in mitochondrial β-oxidation of fatty acids was found in the liver of TCC exposed female offspring (Fig 6B); PPARα (0.59 fold, *p*<0.05), CPT1A (0.31 fold, *p*<0.001) and CPT2 (0.58 fold, *p*<0.05) were all significantly lower in TCC females relative to their controls. We also observed significant down regulation for leptin (0.50 fold, *p*<0.05) and adiponectin (0.43 fold, *p*<0.001) in adipose tissue (Fig 6A). There was no significant change in mRNA expression levels in any of the measured genes from the male offspring.

We subsequently investigated whether or not these changes in mRNA expression were reflected in changes to hepatic triglycerides in TCC exposed offspring (Fig 6C). Consistent with the gene expression results, a three-fold increase in hepatic triglycerides was found in TCC-exposed female offspring (93.63 mg/dL ± 7.97, *p*<0.001) relative to controls (29.41 mg/dL ± 9.64). No significant changes in hepatic triglycerides were noted between groups for male offspring.

## Discussion

The present study provides evidence that maternal exposure to concentrations of TCC comparable to levels found in the US water supply causes measurable effects in exposed offspring. We have shown that exposure to an environmentally relevant concentration of TCC results in TCC crossing the placental barrier and being transferred to the developing fetus. Additionally, exposure of offspring solely through breastfeeding during lactation, was shown to promote an increase in offspring body weight and widespread biodistribution of TCC-related compounds. Significant weight gain persisted throughout maturity even after TCC exposure through lactation was withdrawn. Neonatal exposure to TCC through breast milk also resulted in TCC distribution and accumulation in organs involved in lipid metabolism, such as the brain, fat, heart and muscle. This bio-distribution differed from the TCC exposed dams, in which major organs of the reticuloendothelial system such the lung, liver, heart and spleen contained higher concentrations of TCC and its metabolites. We observed a dramatic difference in total recovered ^14^C in brain tissue for offspring (14–18%) when compared to dams (<1%). The developing brain has been shown to be more susceptible to toxin exposure compared to adults due to increased blood-brain barrier (BBB) permeability [26, 27]. Therefore, higher total recovered ^14^C in the brain tissue after neonatal exposure may have been a result of increased BBB permeability. Although a higher percentage of recovered ^14^C was in brain tissue for exposed offspring, dams exhibited a greater decrease in tissue mass compared to offspring suggesting greater toxicity in brain tissue in dams. This is likely due to the differences in TCC dose for dams and offspring; TCC dams were exposed to 100nM TCC, whereas offspring on average were exposed to a much lower dose, which equated to ~1nM TCC-related compounds.

While both TCC exposed male and female offspring demonstrated increases in weight gain throughout maturity, we found that female offspring were more susceptible. The higher weight gain in female offspring (compared to males) was accompanied by significant downregulation in genes involved in energy homeostasis and lipid metabolism. Leptin and adiponectin both stimulate PPARα gene expression and fatty acid oxidation by activating AMP-activated protein kinase (AMPK) [22, 28]; AMPK is expressed in many tissues with high expression of subunits (α1,γ2) in tissues that regulate lipid metabolism and energy homeostasis, such as the heart, muscle, brain and liver [22]. In liver, PPARα, CPT1A and CPT2 are involved in β-oxidation of fatty acids. Potential disruption of fatty acid metabolism for female offspring was further supported by a three-fold increase in hepatic triglycerides. Collectively, these data indicate that lipid metabolism may be affected for TCC exposed female offspring as suggested by the downregulation of genes involved in β-oxidation of fatty acids in adipose and liver tissue.

Our findings showing effects for female offspring after TCC exposure are in agreement with other perinatal exposures to endocrine disrupting compounds (EDC) with estrogenic activity such as bisphenol A (BPA) and diethylstilbestrol (DES), which have been shown to significantly affect female offspring and lipid metabolism after low dose exposures. Exposure to BPA (~4 μM) during gestation and lactation in rats resulted in higher body and adipose tissue weights with increased adipogenesis in only female offspring [25]. Neonatal exposure to DES (ng/day) also resulted in increased offspring weight and body fat in exposed female offspring at six weeks of age with elevated levels of leptin, adiponectin, IL-6, and insulin in serum after exposure [14]. TCC has been shown to have estrogenic activity *in vitro* [11] and is structurally similar to other estrogenic compounds such as triclosan, which may explain why female offspring were more affected than males in our study. While shown to have estrogenic activity *in vitro*, we did not observe an increase in uterine weight for our TCC exposed female offspring in this study. This could be due to several factors; in addition to dose concentration, route of exposure and species or strain may influence uterotrophic responses [29, 30]. Additionally, uterine fluid imbibition is a result from estrogen exposure and care must be taken to preserve the integrity of the uterine tissue to avoid fluid loss during collection [31, 32]. The uterotropic effects of TCC were not the focus of this study, therefore, loss of fluid may have occurred during uterine tissue removal.

Early life exposure to EDCs such as TCC has the potential to cause irreversible outcomes due to the fragile nature of organ systems and protective mechanisms in developing organisms. In this study, we did note differences in TCC bio-distribution in exposed developing offspring when compared to TCC exposure as an adult, which further highlights the need to evaluate the risk of early life exposure to chemicals such as TCC. Taken together, these results are of great relevance given the documented presence of TCC in wastewater and its observed endocrine disruption capabilities. Future studies are needed to fully evaluate the long-term health effects of TCC exposure during development.

## Supporting information

S1 Fig TCC loss in custom made water bottles over two weeks. (TIFF)

S1 Appendix Hierarchical mixed effect statistical model. (DOCX)
