# Supplementary figures and images for: Maternal exposure to an environmentally relevant dose of triclocarban results in perinatal exposure and potential alterations in offspring development in the mouse model
Source: PLoS One. 2017 Aug 9;12(8):e0181996. doi: 10.1371/journal.pone.0181996 (PMC5549899; doi:10.1371/journal.pone.0181996)

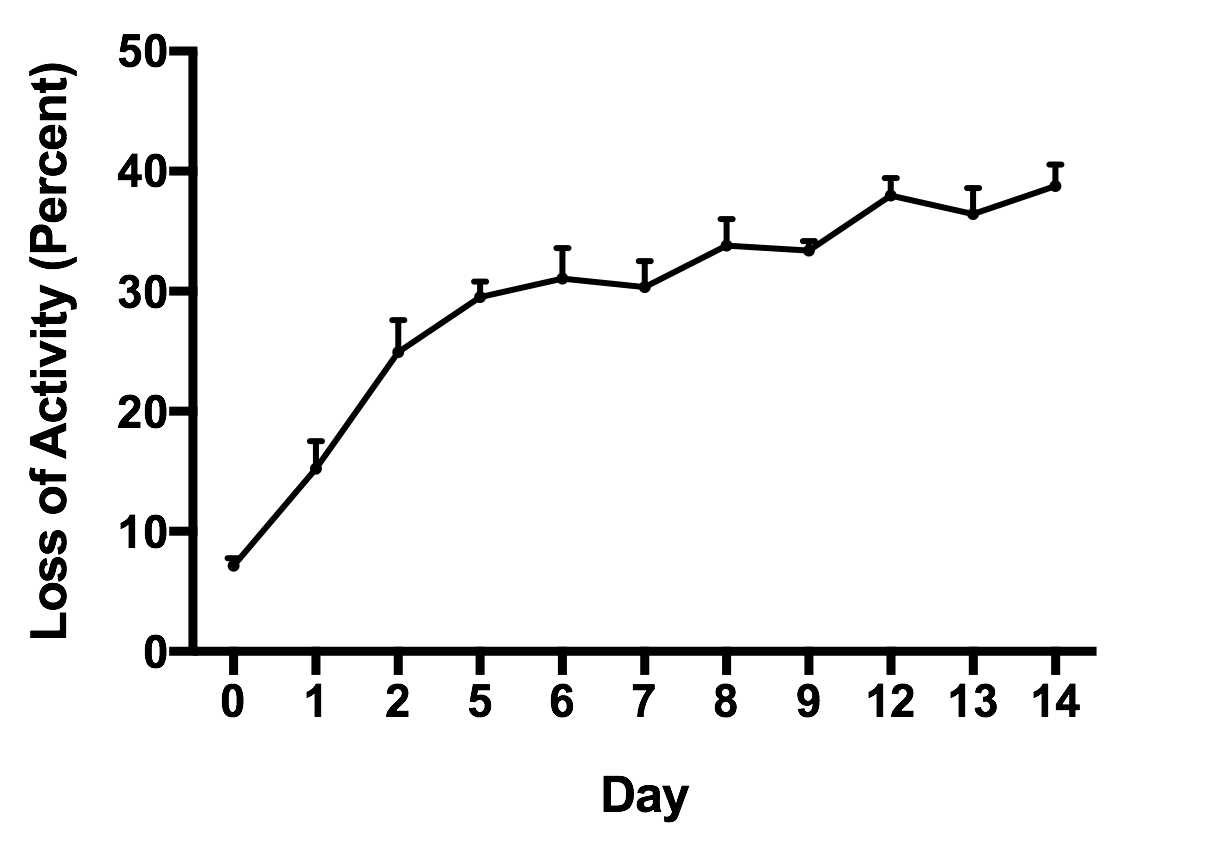

Supplement: S1 Fig — (TIFF) [file pone.0181996.s001.tiff]
