# Supplementary material for: Maternal exposure to an environmentally relevant dose of triclocarban results in perinatal exposure and potential alterations in offspring development in the mouse model
Source: PLoS One. 2017 Aug 9;12(8):e0181996. doi: 10.1371/journal.pone.0181996 (PMC5549899; doi:10.1371/journal.pone.0181996)
Supplement: S1 Appendix — (DOCX) [file pone.0181996.s002.docx]

**S1 Appendix**

The hierarchical mixed effect statistical analysis accounts explicitly for the relationships of variables within offspring- and litter-specific levels, as well as for the influence of litter-level variables on offspring-level ones. The choice of statistical analysis was motivated by the nested structure of the data (repeated measurements were taken at various time points for the each offspring, each of which belongs to one litter, which in turn has its own characteristics, such as litter size and dam group assignment, so that offspring within the same litter are likely correlated with each other due to shared environmental factors). Commonly used alternatives to mixed effect statistical models are repeated measures ANOVA and usage of litter averages as the experimental unit [1, 2]. However, given the facts that 1) sample sizes were smaller after PND42 (due to offspring being sacrificed for other measurements), 2) using litter averages (as opposed to weights of individual offspring) would result in loss of information, and 3) in addition to hypothesis tests regarding the presence or absence of the effect, we are also interested in effect sizes, hierarchical mixed effect model was the natural statistical approach for this analysis. Formally, the weight *y_ijkl_* at time point *l* of offspring *k* from litter *j* whose dam was assigned to treatment *i is:*

*y_ijkl_* ∼ *α*_0_*_i_* + *α*_1_*δ*_0_*_ijk_* + *a*_0_*_k_*_(_*_i_*_,_*_j_*_)_ + (*β*_0_ + *β*_1_*δ*_0_*_ijk_* + *β*_2_*δ*_1_*_l_*)*t_l_* + *ϵ_ijkl_* (1)

where *α*_0_*_i_* is the population group-specific intercept for group *i*, *α*_1_ is a male-specific contribution to the intercept, and *δ*_0_*_ijk_* is an indicator function taking value 0 if the offspring *ijk* is female, and 1 if it is male. The offspring- and offspring-within-litter-level nested random intercepts are captured in *a*_0_*_k_*_(_*_i_*_,_*_j_*_)_ (which can be broken into the offspring- and litter-specific random intercepts, *a*_1_*_ij_* and *a*_2_*_ijk_*, respectively). *β*_0_ captures the overall population slope (that is, the rate of weight change over time), *β*_1_ represents the male-specific additional contribution to the slope, and *t_l_* is the postnatal day at time point *l*. Finally, the change-point of the response trajectory is modeled with *β*_2_*δ*_1_*_l_t_l_*, where *β*_2_ is the effect-size of the change in slope that takes place after postnatal day 30, with *δ*_1_*_l_* taking value 0 if *t_l_* < 31 and 1 if *t_l_* ≥ 31. *ϵ_ijkl_* encompasses the residual variance in the model due to unaccounted random effects, such as measurement error. Slopes for the fixed effect group as well as for both random effects, litter and offspring, were considered. However, they were not significant, and hence were not included in the model. Because offspring sex was only determined at PND21, pre- and post-PND20 weight data are analyzed separately. The model for data prior to PND20 differs from that of data post PND20 in that it does not include the change point term, *β*_2_*δ*_1_*_l_t_l_*, nor does it include the term to account for sex, *β*_1_*δ*_0_*_ijk_*, and it includes a slope contribution, *β*_3i_, for fixed effect for group:

*y_ijkl_* ∼ *α*_0_*_i_* + *α*_1_*δ*_0_*_ijk_* + *a*_0_*_k_*_(_*_i_*_,_*_j_*_)_ + *β*_0_*t_l_* + *β*_3i_*t_l_* + *ϵ_ijkl_* (2)

Tables below display the estimated fixed-effect coefficients, along with their standard errors and p-values for PND1-20 and PND21-56 data, respectively.

**Fixed effect coefficient estimates, standard errors, and p-values from the linear mixed effect model described in (2) for PND1-20. The time multiplied by the slopes is postnatal day minus 20.**

| **Coefficient** | **Estimate** | **Std.Error** | **P value** |
| --- | --- | --- | --- |
| Baseline intercept | 2.41 | 0.24 | 1.13e-20 |
| TCC intercept | 0.20 | 0.33 | 5.69e-01 |
| Baseline slope | 0.39 | 0.01 | 2.15e-150 |
| TCC slope | 0.05 | 0.01 | 2.34e-05 |

**Fixed effect coefficient estimates, standard errors, and p-values from the linear mixed effect model described in (1) for PND21-56.**

| **Coefficient** | **Estimate** | **Std.Error** | **P value** |
| --- | --- | --- | --- |
| Baseline intercept | 11.50 | 0.61 | 7.85e-65 |
| TCC intercept | 2.37 | 0.80 | 1.64e-02 |
| Male intercept | 1.88 | 0.31 | 1.61e-08 |
| Baseline slope | 1.19 | 0.02 | 0.00e+00 |
| Change-point slope | -1.01 | 0.02 | 1.13e-251 |
| Male slope | 0.21 | 0.01 | 1.38e-105 |

**References**

1. Lazic SE, Essioux L. Improving basic and translational science by accounting for litter-to-litter variation in animal models. BMC neuroscience. 2013;14:37.

2. Wainwright PE, Leatherdale ST, Dubin JA. Advantages of mixed effects models over traditional ANOVA models in developmental studies: a worked example in a mouse model of fetal alcohol syndrome. Developmental psychobiology. 2007;49(7):664-74.
